# An unusual case of Behçet's disease presenting with postpartum ovarian iliac vein thrombosis and pulmonary embolism

**DOI:** 10.1186/1477-9560-4-20

**Published:** 2006-12-09

**Authors:** Sonia Hammami, Mondher Golli, Faouzi Addad, Chiraz Hafsa, Amira Hamzaoui, Silvia Mahjoub, Amor Gannouni

**Affiliations:** 1 Department of Internal Medicine, University Hospital 'F. Bourguiba' Monastir, Tunisia; 2 Department of Radiology, University Hospital 'F. Bourguiba' Monastir, Tunisia; 3 Department of Cardiology, University Hospital 'F. Bourguiba' Monastir, Tunisia

## Abstract

Thrombosis of the ovarian vein is a rare complication which arises classically in the postpartum. We report a case of 24-year-old woman with a history of Behçet's disease, who presented with pelvic and thoracic pain, tachycardia, dyspnea and fever occurring 2 weeks after delivery. Computed tomography revealed an ascending thrombosis of the iliac and right ovarian veins complicated by bilateral pulmonary embolism. The patient responded well to the combination of anticoagulants and immunosuppressive agents. Behçet's disease should also be considered as an etiologic factor for ovarian vein thrombosis.

## Background

Behçet's disease (BD) is a multisystemic recurrent inflammatory disorder. First described was the characteristic triad of oral ulcerations, genital ulcerations and, ocular lesions and later additional manifestations were described, including disorders of the skin, joints, large vessels, lungs, brain, and gastrointestinal and genitourinary tracts [1]. BD strongly predisposes patients to venous and arterial thrombosis but pulmonary thromboembolism is very uncommon and it is occasionally observed in the form of deep vein thrombosis [2].

We present a patient with BD who developed thrombosis of the ovarian and iliac veins complicated by pulmonary thromboembolism during the postpartum period.

## Case report

**Figure 1:**
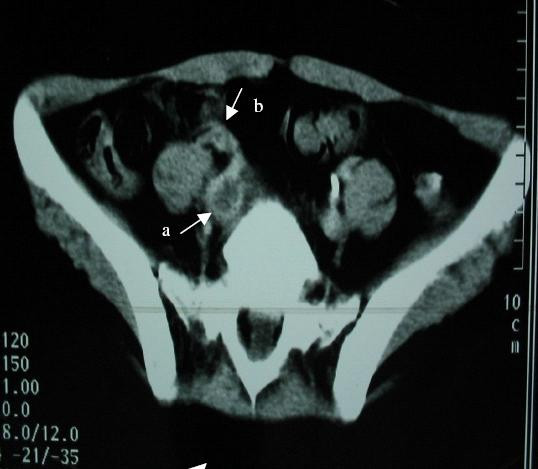
Abdominal computed tomographic scan showing an iliac vein thrombosis (a) and ascending thrombosis of the right ovarian vein (b).

**Figure 2:**
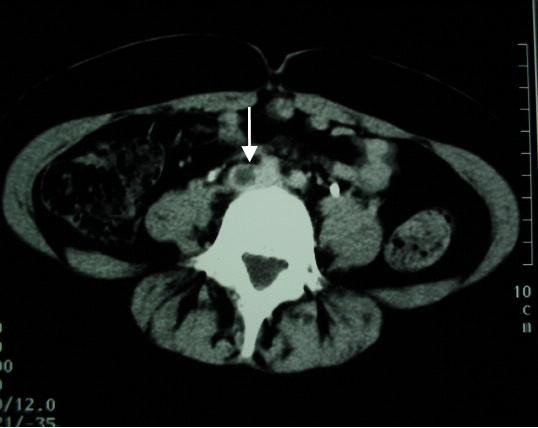
Abdominal computed tomographic scan showing a common iliac vein thrombosis.

A 24-year-old Tunisian woman was admitted to the hospital 2 weeks after vaginal delivery with a 10-day history of pain in the right leg, right iliac fossa, and flank. In the following days, these symptoms worsened. The patient developed chest pain, tachycardia, dyspnea, and fever increasing in severity. She was unresponsive to antibiotic therapy. A detailed past medical history revealed that she had no risk factors, but had been diagnosed with BD 2 years before with recurrent oral and genital aphthous, pseudofolliculitis, and erythema nodosum in the lower extremities. Colchicine therapy had been started, but after a while the patient stopped the therapy on her own. Physical examination upon admission revealed a fever of 38.5°C. She also had oral ulcers on the lips, hypopigmented genital scars, and pseudofolliculitis. Heart sounds and bilateral lung areas were normal upon auscultation. Laboratory tests were consistent with an inflammatory condition with a high erythrocyte sedimentation rate of 130 mm in the first hour, a C reactive protein level of 78.3 mg/dl (normal range 0–5 mg/dl), a hemoglobin value of 11 g/dl and a leukocyte count of 5400/mm^3 ^with a normal differential count. Urinalysis, and liver and kidney function were normal; antinuclear antibody tests were negative ; platelet count, protein C, protein S, and antithrombin III levels were within normal limits ; and anticardiolipin antibodies and lupus anticoagulant were absent. Her plasma total homocysteine level was 17 μmol/l (normal < 10 μmol/l). Chest radiography and electrocardiogram were normal with the exception of sinus tachycardia. Color Doppler echography and abdominal computed tomographic scan (CT) revealed an ascending thrombosis of the iliac and right ovarian veins (Figs. 1 and 2). Chest helical CT revealed a bilateral mural thrombus of segmental pulmonary arteries and a triangular area of opacity representing a pulmonary infarct at the posterior segment of the right lower lobe. The patient was treated with anticoagulants, 1 g of methylprednisolone per day for 3 days, then 1 mg/kg/day orally before tapering gradually, colchicine 1 mg/day, and six monthly pulses of intravenous cyclophosphamide followed by azathioprine at a dose of 2.5 mg/kg/day. The patient's symptoms diminished. One year later, the patient was asymptomatic and color Doppler ultrasonography revealed no evidence of ovarian or iliac vein thrombosis.

## Discussion

The influence of pregnancy on BD is variable and has not yet been clearly demonstrated. Pregnancy is associated with disease exacerbation in 27 % of patients [3]. Our patient had active BD when she became pregnant for the second time (ulceration of the mouth and pseudofolliculitis), and simultaneously developed two thrombotic events at different sites during the puerperium. This suggests a possible interaction between BD and pregnancy.

Postpartum ovarian vein thrombosis (POVT) is an uncommon but potentially serious disorder. Melis et al reported that the risk of deep vein thrombosis is elevated 5 to 6 fold during pregnancy and 2 to 3 fold during the puerperium [4]. This hypercoagulable state is present for 6 weeks postpartum, and can be explained by venous stasis or changes in the hemostatic system with increasing procoagulant factors and platelet adhesiveness. In addition, intrauterine infection and puerperal hypercoagulability, due to recent surgery, malignancy, thrombophilias, lupus, sickle cell disease, Crohn's disease, obesity, fluid and electrolyte imbalance, etc., might also be contributing factors [5-7]. Salomon et al reported that 50 % of patients have an inherited thrombophilia [8]. Our patient did not have symptoms suggestive of any of these disorders. In addition, a diagnosis of intrauterine infection was not supported with complete clinical resolution after anticoagulant and immunosuppressive therapy. Vasculitic and thrombotic abnormalities of coagulation or fibrinolitic activity are both well known to predispose to thrombosis in BD [9]. Additionally, in this case hyperhomocysteinemia might have contributed to the hypercoagulable state. Ates et al [10] reported that homocysteine levels are independently associated with thrombosis, but recent studies did not reveal such an association [11].

The main sign of typical clinically significant ovarian vein thrombosis is pain in the flanks or the iliac fossa, in association with fever. Thrombosis is most frequently identified in the right ovarian vein due to the physiologic dextrorotation of the uterus, which might compress the ovarian vein. When the thrombus is located on the right side, several conditions should be considered in the differential diagnosis, such as pyelonephritis and acute appendicitis. Besides the clinical signs, CT and magnetic resonance imaging are recommended as the procedures of choice for ovarian vein imaging [12]. They are also recommended for evaluating wall thickening of the pulmonary artery.

POVT is a rare complication with potentially devastating consequences, including caval thrombus, pulmonary thromboembolism, and death [13]. In BD, however, pulmonary embolism rarely occurs because the thrombi in the inflamed veins are strongly adherent. It is therefore possible that in active BD, pulmonary involvement is in part a result of insitu pulmonary pathology rather than embolization from a systemic vein. In Mogulkoc's review, 6 of 12 patients with pulmonary arterial thrombosis were free of peripheral thrombi [14]. The incidence of pulmonary embolism in women with POVT has been reported to be from 13 % to 33 % especially when thrombus may extend into inferior vena cava [13]. Kettaneh et al. report two patients with POVT complicated by pulmonary embolism in both cases [15]. In our case pulmonary embolism may be a complication of the iliac vein thrombosis, there is no extension into vena cava of the ovarian vein thrombosis. Our patient responded well to the combination of anticoagulant, colchicine, corticosteroids and immunosuppressive agents. There is no agreement on the therapy of deep venous thrombosis and pulmonary embolism in BD. Thrombus is supposed to be due to the vasculitic process and the aim of our treatment being to eradicate the thrombus and prevent recurrence, for this reason immunosuppressant therapy can be added to anticoagulation in active Behçet's disease associated central venous thrombus especially when biological inflammation is present, On the other hand it is important to note that in the presence of pulmonary involvement, anticoagulant alone failed to prevent recurrence in some patient. Surgical treatment for this condition is generally reserved for patients for whom anticoagulation is contraindicated and who have recurrent pulmonary emboli despite medical management.

To the best of our knowledge, this is the first case of postpartum ovarian thrombosis in BD associated with hyperhomocysteinemia and pulmonary embolism. We conclude that BD should always be considered as an etiologic factor for ovarian thrombosis, especially during pregnancy and puerperium, for this reason prophylactic anticoagulant therapy might be considered depending on the risk factors.

## Abbreviations

BD: Behçet's disease

CT: tomographic scan

POVT: Postpartum ovarian vein thrombosis

## Competing interests

The author (s) declares that they have no competing interests.

## Authors' contributions

S.H. : conceived of the study, tacked out the responsibility for diagnosis of patient, their evolution and elaborated in the design of this case and draft the manuscript ; M G : tacked out computed tomographic scan, ; F. A : interested in the cardiac and pulmonary aspects; ; CH : tacked out X-ray, colour and Doppler ultrasonography examinations AH : interested in the hospital evolution. S. M. tacked out the responsibility for diagnosis of patient, and helped to draft the manuscript; A G: revised the article critically for important intellectual content and have given final approval of the version to be published.

All authors read and approved the final manuscript.
